# The inhibitory action of camel urine on human platelet surface glycoproteins could be the potential mechanism for its reported anticancer effects

**DOI:** 10.3389/fphar.2026.1874341

**Published:** 2026-09-17

**Authors:** Abeer Khalid Al-Ghumlas, Maha Hamadien Abdulla, Mohammad S. El-Wetidy, Abdel Galil Mohammad Abdel Gader

**Affiliations:** 1 The Coagulation Research Laboratory, Physiology Department, College of Medicine, King Saud University, Riyadh, Saudi Arabia; 2 Colorectal Research Chair, Department of Surgery, College of Medicine, King Saud University, Riyadh, Saudi Arabia; 3 College of Medicine Research Center, King Saud University, Riyadh, Saudi Arabia

**Keywords:** aspirin, camel urine, cancer therapy, CD41, CD61, flow cytometry, platelet glycoproteins

## Abstract

**Background:**

Numerous medical properties have been attributed to camel urine (CU), including anticancer activity as reported in the literature. Laboratory studies have also demonstrated that CU inhibits platelet aggregation in a manner similar to aspirin (acetylsalicylic acid, ASP), a well-known antiplatelet agent with documented anticancer relevance. It is therefore of interest to further explore similarities in the actions of these agents using molecular approaches. This study aimed to characterize the effect of CU on the expression of human platelet GP receptors (CD41, CD61, CD42a, and CD42b) and to compare these effects with those of aspirin, a well-established antiplatelet agent.

**Methods:**

The effect of CU on human platelet aggregation in response to adenosine diphosphate (ADP) and arachidonic acid was evaluated using light transmission aggregometry. CU that showed an inhibitory effect on platelet aggregation was subsequently used for flow cytometric analysis, and ASP was used for comparison as a positive control. Platelet rich plasma was prepared from human blood, and washed platelets were subsequently isolated. The washed platelets were incubated with fresh CU (9.1% v/v; n = 8) or ASP (stock concentration: 81 mg/mL n = 6) for 5 min at room temperature, while parallel untreated samples served as controls. Platelet GP receptor expression was subsequently evaluated by flow cytometric quantification of CD61, CD41, CD42a, and CD42b using a BD FACSCalibur and BD FACSCanto II.

**Results:**

Flow cytometric analysis revealed that both CU and ASP caused a significant reduction in the expression of CD61, CD41, CD42a, CD42b, and the CD41/CD61 complex compared with untreated samples. However, ASP exhibited a stronger inhibitory effect than CU. ASP induced greater inhibition of CD41 (48.3%) followed by CD61 (41.5%), whereas CU resulted in 1: 30.6% and 28.2% inhibition of CD61 and CD41, respectively.

**Conclusion:**

These findings indicate that CU possesses antiplatelet activity, which may warrant further investigation as a potential contributor to its reported anticancer effects.

## 1 Introduction

The one-humped camel (*Camelus dromedarius*) has long been valued as a source of food and transportation in desert regions. In addition to its nutritional importance, camel milk and urine have been used in traditional medicine for centuries (Gader and Alhaider, 2016; Gole and Hamido, 2020). Increasing scientific evidence has reported potential biological activities of CU and camel milk in cancer (Alebie et al., 2017; Romli et al., 2017; Gupta et al., 2021; El-Kattawy et al., 2021; Baothman et al., 2024), chronic hepatitis B and C (Saltanat et al., 2009; Mohamed et al., 2015; El-Fakharany et al., 2017; Hosseini et al., 2017; Khan et al., 2021), peptic ulcers disease (Al-Lbban, 2024), and diabetes (Ashraf et al., 2021; Fallah et al., 2020; Shaban et al., 2022). These observations have stimulated interest in investigating the underlying mechanisms responsible for their reported therapeutic effects.

Previous studies from our laboratory investigated the unique biology of the camel haemostatic system, with particular focus on camel platelet structure and function (Hussein et al., 1992; Abdel Gader et al., 2006; Abdel Gader et al., 2008; Al Ghumlas and Gader, 2013) and identified distinctive features; for example, camel platelets have shown markedly inhibited aggregation in response to various agonists including ADP and arachidonic acid (AA) when compared with human platelets. Further studies demonstrated that both camel plasma and urine (Al-Ghumlas, 2009; Alhaidar et al., 2011; Al Yahya et al., 2016; Al-Ghumlas, 2020) inhibited the human platelets aggregation responses to ADP and AA in a dose-response manner. This inhibitory effect of CU resembled that of aspirin (acetylsalicylic acid; ASP), as evidenced by inhibition of AA-induced platelet aggregation, and that of clopidogrel by inhibiting ADP-induced platelet aggregation.

Accumulating evidence indicates that activated platelets contribute to tumor growth, angiogenesis, immune evasion, and metastasis through their interactions with circulating tumor cells (Bambace and Holmes, 2011; Wojtukiewicz et al., 2015; Gindri dos Santos et al., 2026). In fact, platelet glycoproteins, including CD41, CD42a, CD42b and CD61, play important roles in platelet adhesion, aggregation, tumor progression, and metastasis (Martins Castanheira et al., 2022; Yap et al., 2019; Rodriguez-Martinez et al., 2022). Several studies have reported that inhibition of platelet function or platelet-associated glycoproteins can reduce tumor growth, metastasis, and improve responses to anticancer therapies (Mitrugno et al., 2017; Shiao et al., 2017; Wojtukiewicz et al., 2017). Consequently, inhibition of platelet activation has been proposed as one mechanism that may contribute to the reported anticancer effects of antiplatelet agents, particularly ASP which is widely reported to have the potential to prevent cancer, reduce tumor growth, metastasis in addition to prolonging the survival of cancer patients (Choe et al., 2012; Wojtukiewicz et al., 2017; Xie et al., 2021; Johns et al., 2023; Ma et al., 2023; Pandey et al., 2024).

Based on these observations, we hypothesized that the antiplatelet activity of CU may represent one potential biological mechanism underlying its previously reported biological activities, including its reported anticancer effects (Al-Yousef et al, 2012). Although CU has been shown to inhibit platelet aggregation and has been suggested to have potential anticancer properties, the molecular basis of these effects remains unknown, particularly whether they are mediated through modulation of platelet surface glycoprotein (GP) receptors. Therefore, the present study aimed to characterize the effects of CU on the expression of human platelet GP receptors (CD41, CD61, CD42a, and CD42b) and to compare these effects with those of aspirin, a well-established antiplatelet agent. This study aims to provide mechanistic insight into the antiplatelet activity of CU. Any implications of these findings for the previously reported anticancer effects of CU remain speculative and require further experimental confirmation.

## 2 Materials and methods

### 2.1 Study design

#### 2.1.1 Blood collection and processing

Blood samples were obtained from healthy volunteer blood donors at the blood bank at King Khalid Hospital, King Saud University (KSU). Informed consent for blood donation was obtained by the blood bank at the time of collection. All samples were anonymized and used in accordance with institutional ethical approval. The study protocol was approved by the Institutional Review Board of the College of Medicine, King Saud University (KSU). The blood samples were transferred without delay (within 2 h of collection) to the coagulation research laboratory.

#### 2.1.2 Camel urine collection

Fresh urine samples were collected individually from 35 healthy female dromedary camels (*C. dromedarius*), aged 2–10 years, from a camel breeding farm outside Riyadh city. The animals were maintained under routine veterinary supervision, were clinically healthy, and had free access to water and standard camel feed. The inclusion criteria were female dromedary camels aged 2–10 years with no apparent clinical signs of disease. Camels showing signs of illness or other abnormal health conditions at the time of sample collection were excluded. Urine samples were not pooled and urine collection was performed during feeding by experienced camel attendants as previously described (Alhaidar et al., 2011). Urine was allowed to flow directly into sterile stainless-steel containers before transfer to sterile glass vials. Samples were transported to the Coagulation Research Laboratory, Physiology Department, College of Medicine, King Khalid University Hospital, for subsequent platelet aggregation and flow cytometric analyses within 4 h of collection. All urine samples were initially evaluated for their effects on platelet aggregation and then stored at −80 °C until further use in the flow cytometry experiments. Based on the aggregation results, eight urine samples that exhibited inhibitory activity were subsequently selected for flow cytometric analysis of platelet glycoprotein receptor expression. To minimize potential inter-animal variability, all urine samples were collected from animals maintained on the same farm under similar environmental, dietary, and management conditions. Urine samples were collected individually and were not pooled. In addition, the initial aggregation experiments allowed us to ascertain the antiplatelet activity of individual CU samples and select those demonstrating inhibitory activity.

### 2.2 Platelet aggregometry

#### 2.2.1 Effect of camel urine on human platelets aggregation

The human blood samples were centrifuged at 1,000 rpm for 7 min to separate platelet rich plasma (PRP), which was used for the platelet aggregation studies. We preincubated human PRP with 0.05 mL of raw CU for 2 min. Aggregation was measured in response to ADP (20 μmol/L) and AA (1.64 mmol/L), (Bio/Data Corp. United States), as detailed before (Abdel Gader et al., 2006). All these concentrations of agonists represent the final concentrations obtained by adding 20 μL of the aggregating agent to 180 μL of PRP. Aggregation was recorded in an Aggregation Profiler (PAP4, Bio/Data, United States), which registers the results of the aggregation responses as maximum aggregation (MA%). Camel urine that showed platelet aggregation inhibitory action on human PRP was used for the flowcytometric studies.

### 2.3 The flow cytometry studies

#### 2.3.1 Preparation of the platelets

The first part of the procedure includes isolation and washing of platelets as described elsewhere (Linden, 2013). PRP is diluted with citrate wash buffer 1:8 and then centrifuge for 10 min at 1,200×g at room temperature. The supernatant is aspirated and the pellet is resuspended gently in citrate wash buffer (without vortex). The wash is repeated and the washed platelet is resuspended in 4-(2-hydroxyethyl)-1-piperazineethanesulfonic acid (HEPES)-buffered Tyrode’s solution.

#### 2.3.2 Treatment with camel urine and/or aspirin

The second part of the procedure includes dividing the washed platelet suspension into two aliquots. One aliquot was incubated with 50 µL of fresh CU; n = 8 or 50 µL of ASP solution; n = 6 for 5 min at room temperature, while the second aliquot was left untreated and served as the control. CU was added at a fixed volume of 50 µL to 500 µL of washed platele suspension, resulting in a final concentration of approximately 9.1% (v/v). For the ASP-treated samples, a stock solution was prepared by dissolving one tablet (81 mg) of Aspirin (Julphar, Ras Al Khaimah, United Arab Emirates) in 1 mL of distilled water (stock concentration: 81 mg/mL). Subsequently, 50 µL of the stock solution were added to 500 µL of washed platelet suspension. The aspirin concentration used in the present study was used with reference to a previously published *in-vitro* approach (Khosla et al., 1982). All experiments were conducted using the same platelet preparation protocol, and treated and control samples were processed in parallel under identical experimental conditions.

#### 2.3.3 The glycoprotein specific monoclonal antibodies

The following glycoprotein specific monoclonal antibodies were employed:

CD41a (BD Pharmingen, Cat. 555467) which is associated with platelet membrane glycoprotein GPIIb, CD61 (BD Pharmingen, Cat. 348093) which recognizes GPIIIa; GPIIb is non-covalently associated with GPIIIa to form complex GPIIb/GPIIIa which is a receptor for fibrinogen fibronectin, von Willebrand factor and vitronectin that plays a major role in platelet aggregation, CD42b (BD Pharmingen, Cat. 555473) which is associated with CD42a (BD Pharmingen, Cat. 348083), CD42c, and CD42d to form the GPIb-V-IX complex, that binds with the von Willebrand factor receptor.

#### 2.3.4 Flow cytometry analysis

Platelets from each sample (treated or non-treated) were divided into six FACS tubes (50 µL/each). Monoclonal antibodies (10 µL) were added according to the following plan: Tube 1: Blank: non-stained, tube 2: FITC Mouse Anti-Human CD61, tube 3: PE Mouse Anti-Human CD41a, tube 4: FITC Mouse Anti-Human CD42a, tube 5: PE Mouse Anti-Human CD42b and tube 6: Mixture (1:1) of CD61 and CD41a. All tubes were incubated in the dark for 20 min at room temperature. The samples were then washed with 2 mL pf 1X PBS and centrifuged at 1,500 rpm for 5 min at room temperature. Then The supernatant was aspirated and cells were fixed with 1 mL of 2% Paraformaldehyde and incubated for 10 min at room temperature in the dark. The effect of CU on platelets GPs was analyzed using a BD FACSCalibur flow cytometer, whereas the ASP treated samples were analyzed using a BD FACSCanto II flow cytometer (Both machines from BD Bioscience, Franklin Lakes, NJ, United States).

### 2.4 Statistical analysis

Statistical analyses were performed with SPSS software version 22.0 (IBM Corp., Armonk, NY, United States). To select the relevant statistical procedures, the data were first assessed for normality with the Shapiro-Wilk test. The Paired Sample t-test was used to compare variables with normally distributed data across paired groups. When data did not follow a normal distribution, the Wilcoxon Signed-Rank Test was used as a nonparametric alternative. We calculated mean ± standard deviation (SD) for all numerical variables. All tests were two-tailed, with statistical significance at a P < 0.05.

## 3 Results

### 3.1 Effect of camel urine on human platelet aggregation

The addition of raw CU to human PRP resulted in a significant inhibition of human platelets aggregation in response to ADP; the average MA% of human platelet aggregation response to ADP was: (Mean ± S.D: 35.8 ± 18.7), which is significantly lower than the control: (Mean ± S.D: 56.2 ± 9.8); P-value <0.05. Similarly, the human platelets aggregation response to AA decreased after the addition of raw CU (Mean ± S.D: 53.3 ± 22.0) as compared with the controls (Mean ± S.D: 62.2 ± 13.6) but this decrease was not statistically significant. Figure 1 showed the MA% of the effect of CU on agonist-induced human platelet aggregation. In addition, dose-response experiments using ADP and AA-induced human platelet aggregation demonstrated a concentration-dependent inhibitory effect of CU (Figure 2).

**FIGURE 1:**
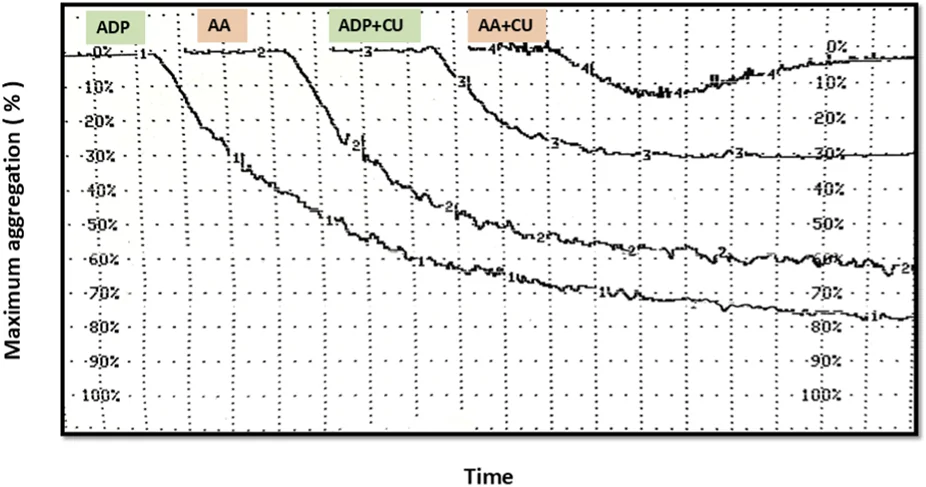
Platelet aggregation tracings demonstrating the effect of camel urine (CU) on agonist-induced platelet aggregation expressed as maximum aggregation percentage (MA%). Human platelet rich plasma (PRP) was stimulated with adenosine diphosphate (ADP) or arachidonic acid (AA) in the absence or presence of neat CU. Traces represent: (1) PRP + ADP; (2) PRP + AA; (3) PRP + CU + ADP; and (4) PRP + CU + AA. Aggregation was measured using light transmission aggregometry.

**FIGURE 2:**
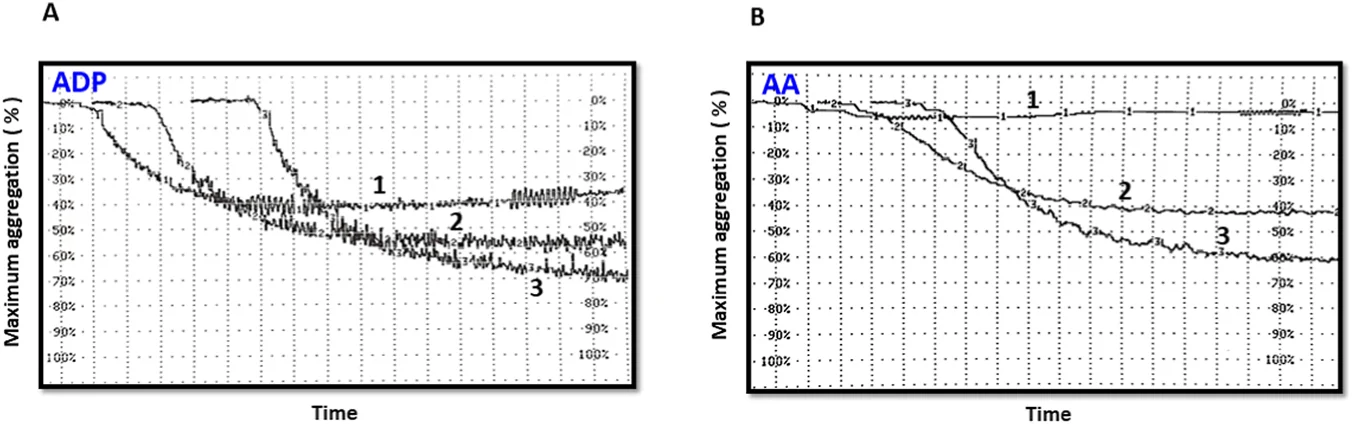
Dose-dependent effect of camel urine (CU) on platelet aggregation. **(A)** ADP-induced platelet aggregation and **(B)** AA-induced platelet aggregation showing the effect of serial dilutions of CU (1:2, 1:4, and 1:8) on human platelet aggregation. Traces 1, 2, and 3 correspond to the 1:2, 1:4, and 1:8 dilutions, respectively.

### 3.2 Effect of camel urine on human platelet expression of CD61, CD41, CD42a CD42b and CD41 + 61

The results of the flow cytometry studies are presented in Table 1 and Figures 3, 4.

**TABLE 1:** Platelets glycoprotein cluster distribution 61 (CD61), cluster distribution 41 (CD41), cluster distribution 42a (CD42 a), cluster distribution 42b (CD42b) and cluster distribution (CD41 + 61 complex) before and after the addition of CU to human platelets.

GP (%)	Before CU (n = 8) Mean ± SD	After CU (n = 8) Mean ± SD	P value	Percentage inhibition
CD61	70.3 ± 9.7	48.8 ± 19.9*	0.005	30.6%
CD41	28.4 ± 14.4	20.4 ± 15.8*	0.004	28.2%
CD42a	72.8 ± 11.4	65.3 ± 13.3*	0.007	10.3%
CD42b	36.0 ± 12.3	29.9 ± 7.4*	0.036	17.0%
CD41 + 61	52.0 ± 13.5	43.4 ± 6.9*	0.048	16.5%

Data are expressed as mean ± SD. *P < 0.05.

**FIGURE 3:**
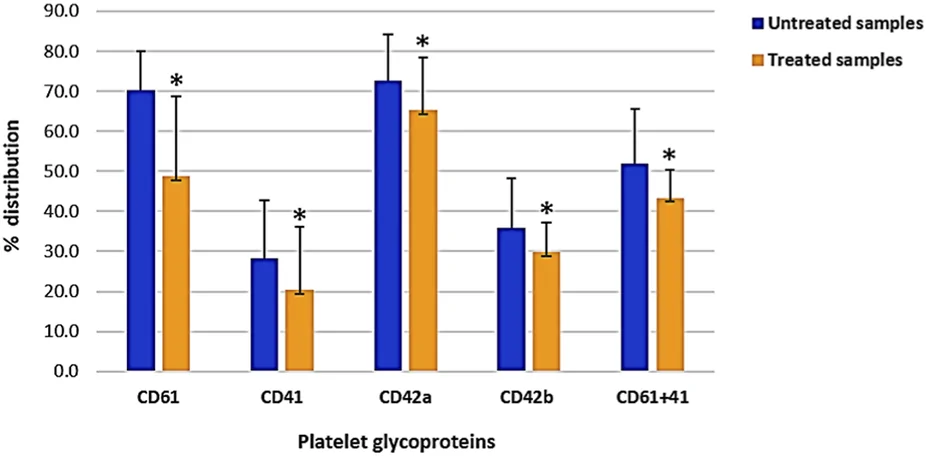
Effect of camel urine (CU) on human platelet glycoprotein (GP) receptors; Platelets cluster distribution 61 (CD61), cluster distribution 41 (CD41), cluster distribution 42a (CD42 a), cluster distribution 42b (CD42b) and the CD41/CD61 complex cluster distribution. Results are shown for CU-treated and untreated (control) samples. *P < 0.05, indicating statistical significance.

**FIGURE 4:**
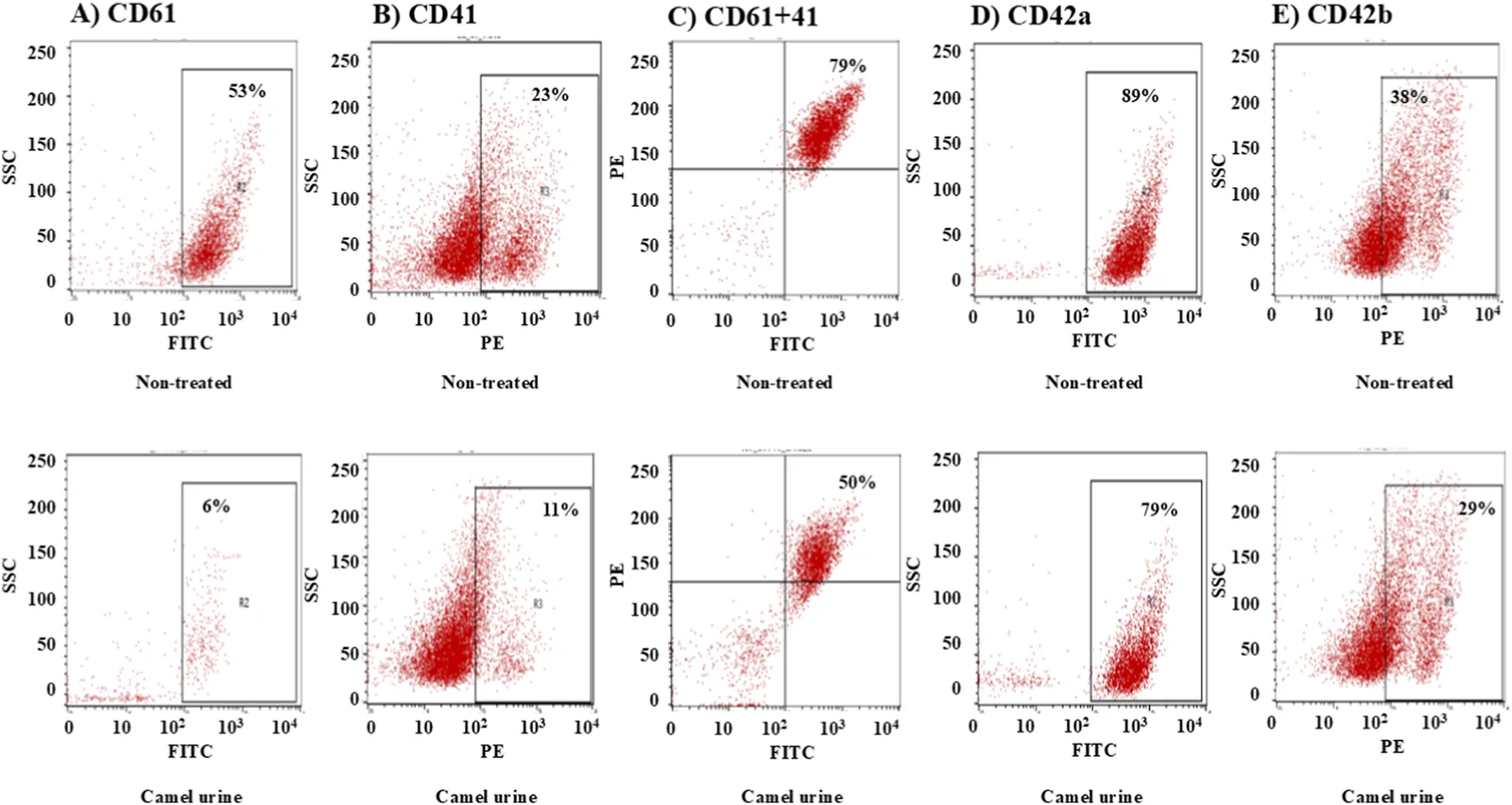
Effect of camel urine (CU) on human platelet glycoprotein (GP) receptors expression. Example of the flow cytometry analysis comparing untreated and CU-treated platelets for the surface expression of **(A)** CD61, **(B)** CD41, **(C)** the CD41/CD61 complex, **(D)** CD42a, and **(E)** CD42b. Data were acquired using a BD FACSCalibur flow cytometer.

#### 3.2.1 Cluster distribution 61 (C61)

In normal platelets extracted from human, the expression of CD61 was (mean ± SD: 70.3% ± 9.7%). However, after incubation with CU, a significant reduction in CD61 expression (mean ± SD: 48.8% ± 19.9%; P < 0.05) was noted.

#### 3.2.2 Cluster distribution 41 (CD41)

CD41 expression showed significant reduction after incubation with CU, when compared to the untreated normal human platelet (mean ± SD: 20.4% ± 15.8% vs.: 28.4% ± 14.4%; P < 0.05).

#### 3.2.3 Cluster distribution 42a (CD42a)

CD42a expression was (mean ± SD: 72.8% ± 11.4%) in untreated normal human platelet, but there was a significant decrease in its expression following the incubation with CU (mean ± SD: 65.3% ± 13.3% P < 0.05).

#### 3.2.4 Cluster distribution 42b (CD42b)

Similar to CD42a, CD42b expression showed a highly significant reduction after the incubation with CU (mean ± SD: 29.9% ± 7.4%) when compared with the normal control levels (mean ± SD: 36.0% ± 12.3% P < 0.05).

#### 3.2.5 Cluster distribution 41 + 61 (CD41 + 61)

Similarly, CD41 + 61 expression showed a significant reduction (mean ± SD: 43.4% ± 6.9%) after addition of CU as compared to the control level before the addition of CU (mean ± SD: 52.0 ± 13.5 P < 0.05).

### 3.3 Effect of aspirin (ASP) on human platelets CD61, CD41, CD42a, CD42b and CD41 + 61 expression

To facilitate comparison of the effect of CU on human platelet glycoproteins expression, ASP which is a well-known antiplatelet agent was used as reference standard that is known to work through different mechanisms including different GP receptors inhibition and the comparison was performed to provide a relative assessment of the inhibitory activity. In the current study, change in the expression of CD61, CD41, CD42a CD42b and CD41 + 61 was observed between the baseline and the samples which were treated with ASP. ASP caused significant reduction in the number of platelets expressing CD61, CD41, CD42a CD42b and CD41 + 61 at the dose of 81 mg when compared with the controls (Table 2; Figures 5, 6). It is of interest to note that more inhibition was observed in the expression of CD41 (48%) followed by CD61 (41.5%) while CU causes 30.6% and 28% inhibition of platelet expressing CD61, CD41 respectively.

**TABLE 2:** Effect of ASP on human platelet glycoprotein receptors; Platelets cluster distribution 61 (CD61), cluster distribution 41 (CD41), cluster distribution 42a (CD42 a), cluster distribution 42b (CD42b) and the cluster distribution (CD41 + 61 complex).

GP (%)	Before ASP (n = 6) Mean ± SD	After ASP (n = 6) Mean ± SD	P value	Percentage inhibition
CD61	97.6 ± 1.3	56.7 ± 21.4	0.028†	41.5%
CD41	68.1 ± 13.4	35.2 ± 14.0*	0.004	48.3%
CD42a	97.4 ± 0.9	58.4 ± 21.5*	0.007	40%
CD42b	81.2 ± 5.4	64.7 ± 14.8*	0.015	20.3%
CD41 + 61	91.7 ± 5.8	62.4 ± 16.0*	0.002	31.9%

Values are presented as mean ± SD., Statistical comparisons were performed using the paired t-test for normally distributed data. †CD61 in the ASP-treated group, which did not meet the assumption of normality, was analyzed using the Wilcoxon signed-rank test. A *P value <0.05 was considered statistically significant.

**FIGURE 5:**
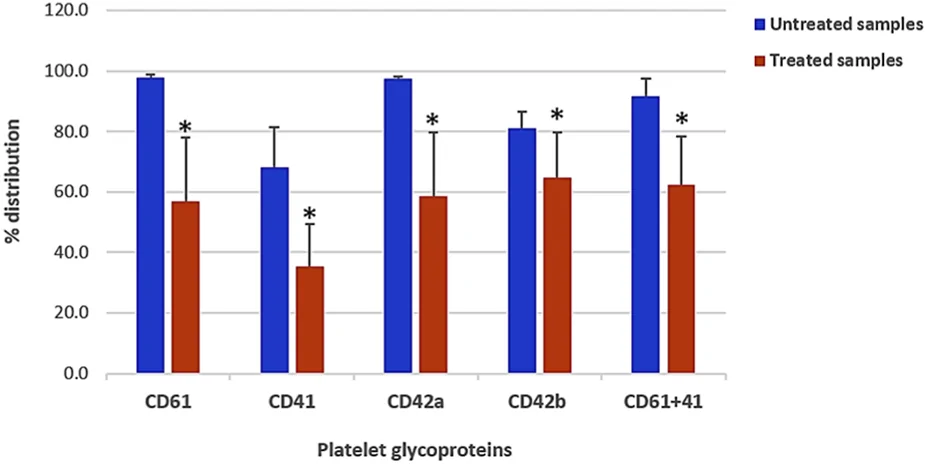
Effect of ASP on human platelet glycoprotein (GP) receptors; Platelets cluster distribution 61 (CD61), cluster distribution 41 (CD41), cluster distribution 42a (CD42 a), cluster distribution 42b (CD42b) and the CD41/CD61 complex cluster distribution. Results are shown for CU-treated and untreated samples (control). Statistical significance was determined by comparison with the control; *P < 0.05, indicating statistical significance.

**FIGURE 6:**
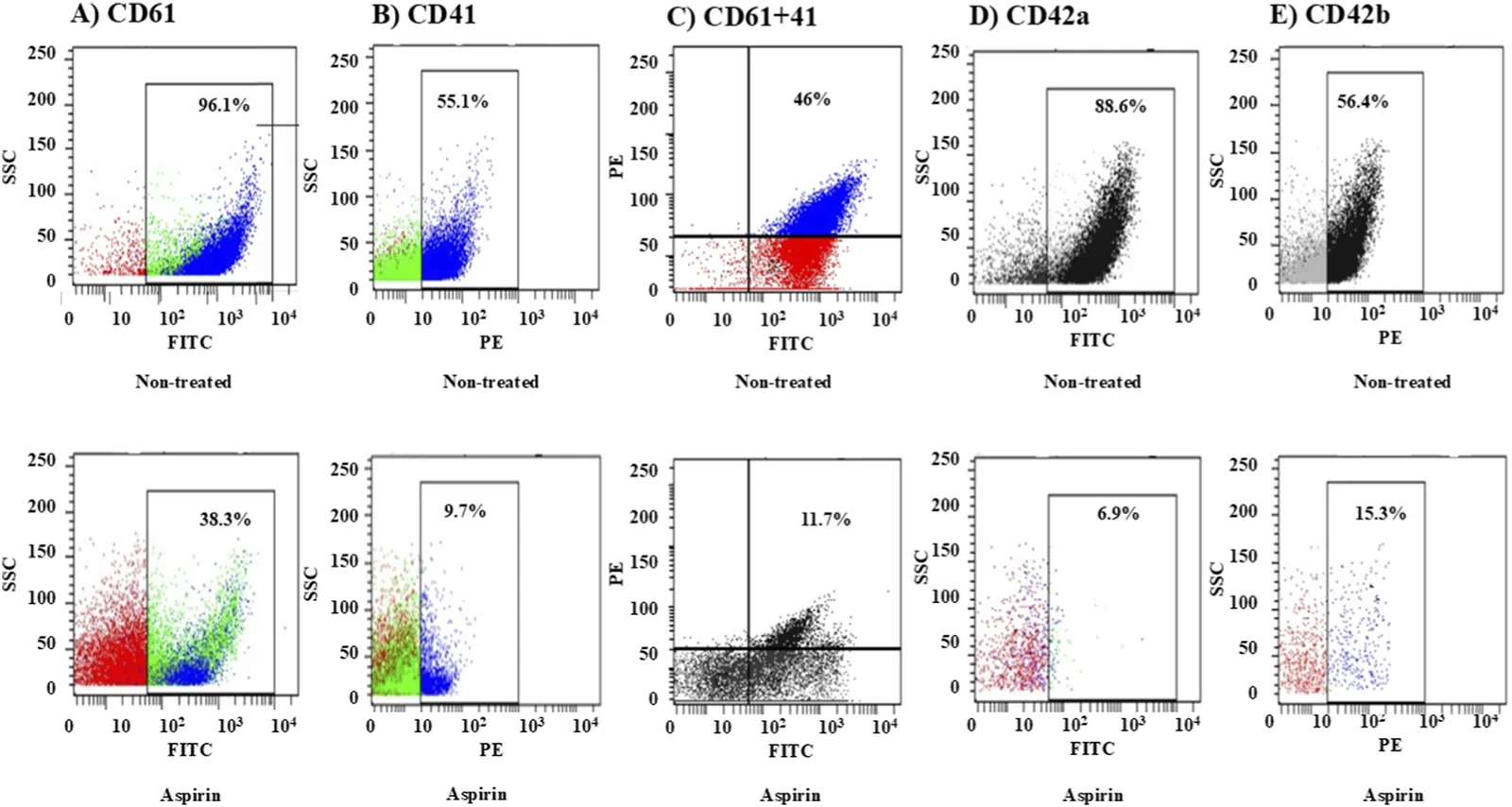
Effect of ASP on human platelet glycoprotein (GP) receptors expression. Example of the flow cytometry analysis comparing untreated and ASP-treated platelets for the surface expression of **(A)** CD61, **(B)** CD41, **(C)** the CD41/CD61 complex, **(D)** CD42a, and **(E)** CD42b. Data were acquired using a BD FACSCanto II flow cytometer.

## 4 Discussion

The prime objective of the current flow cytometry study was to characterize the effect of CU on human platelets surface GP receptors and the results obtained have shown that CU caused significant inhibition of the expression of CD61 (31%), lesser blockage of CD41 (28.2%), CD42a (10.3%), and CD42b (17%). These results provide molecular explanation to the antiplatelet inhibitory action of CU that we have reported before in ADP and AA-induced platelets aggregation responses (Alhaidar, et al., 2011; Al Yahya et al., 2016; Al-Ghumlas, 2020). In these *in vitro* aggregation studies, we have also observed similarity between CU and ASP in their inhibitory action on the platelet aggregation responses to AA. In the current study and like CU, ASP resulted in more significant inhibition of the expression of all the above-mentioned human platelet surface GP receptors CD41 (48.3%), lesser blockage of CD61 (41.5%), CD42a (40%), and CD42b (20.3%). These findings agree with the earlier report of McKenzie et al. (McKenzie et al., 2003). who demonstrated the inhibitory effect of ASP on the surface expression of nine platelet receptors using whole blood flow cytometry.

Although platelets are widely recognized to play a central role in thrombosis and hemostasis, platelets also make major contributions to tumor growth, dissemination, spread and progression, through a wide range of complex physical and functional interactions between platelets and cancer cells. It has been shown that cancer cells can induce not only the activation of platelets and the release of the contents of the platelet granules but also the formation of platelet-tumor aggregates, and platelets may additionally behave as an immune-like cells (Menter et al., 2014; Braun et al., 2021; Desai et al., 2022). For example, *in vitro* incubation of human colon carcinoma cells with human platelets provoked their transformation from epithelial to mesenchymal-like cancer cells that facilitated cancer dissemination (Wojtukiewicz et al., 2015).

The involvement of platelet activation pathways in these processes commences, as expected, at their GP membrane surface receptors. The glycoproteins Ib-IX-V (GP Ib-IX-V, glycoprotein VI (GP VI), and glycoprotein IIb-IIIa (GP IIb-IIIa), as well as protease-activated receptors for thrombin (PAR-1 and PAR-4) are intimately involved in cancer growth and progression (Bambace and Holmes, 2011; Wojtukiewicz et al., 2015). CD61 plays a role in blood flow and cancer development and is involved in the process of angiogenesis. This high-affinity conformation of GPIIb/IIIa on activated platelets can serve as a molecular targeting epitope for the tumor microenvironment in various cancer types in both mice and humans (Yap et al., 2017) and thus inhibition of CD61 expression slows down the growth of cancer and improves its sensitivity to chemotherapy (Yap et al., 2019).

The mounting evidence of the involvement of platelets in cancer disease triggered wide interest in studying the anticancer potential of anti-platelet agents and currently there are numerous reports on the benefits of the anti-platelet therapy in the prevention of cancer, decrease of tumor growth and metastasis in addition to improvement of survival of cancer patients (Wojtukiewicz et al., 2017). Besides, the anticancer action of ASP has been thoroughly investigated both *in vitro* and *in vivo* using cancer cell lines, animal models as well as clinical trials (Guillem-Llobat et al., 2016; Mitrugno et al., 2017; Shiao et al., 2017). ASP may even reduce distant metastases rates, improve disease-free survival, and/or overall survival in cancer patients (Holmes et al., 2010; Rothwell et al., 2011; Lee et al., 2016; Downer et al., 2017; Leader et al., 2017). In addition, the platelet P2Y12 receptor was also shown to control cancer cell/platelet interaction and in this way regulates angiogenesis and cancer progression (Bambace et al., 2010; Schumacher et al., 2013; Wang et al., 2013). Besides, the platelet P2Y12 receptor was shown to mediate tumor cell transendothelial migration and that the P2Y12 inhibitor; ticagrelor inhibits metastasis and improves survival in mouse models of cancer (Schumacher et al., 2013).

With this background information in mind specifically the active involvement of platelets in cancer growth, progression, metastasis and the effect of antiplatelet therapy in controlling all these steps in cancer disease, it is reasonable to take this information to support the long-standing claims of the anticancer action of CU. In the present study, we have shown that CU, like ASP, inhibited the expression of CD41, CD61, CD42a and CD42b. This inhibition will in turn affect platelet activation by disrupting the interaction between activated platelets and tumor cells. The shared inhibition of specific platelet surface GPs observed with CU and ASP may suggest a potential link to the previously reported anticancer effects of CU.

As to the anticancer mechanism(s) of CU, laboratory evidence showed that CU works through cytotoxicity and antiangiogensis. The cytotoxicity and the inhibition of growth and metastasis of cancer by CU was demonstrated in animal studies and cultured cancer cell lines (Alebie et al., 2017; Romli et al., 2017). Other than cytotoxicity, there is also the anticancer mechanism of anti-angiogenesis. Earlier studies from our laboratory have investigated the anti-angiogenic effects of CU using the murine (Swiss albino mice) cannulated sponge implant angiogenesis model assessing both histological and biochemical parameters by measuring key components involved in inflammatory angiogenesis. The results obtained have shown that CU treatment attenuated the main components of the fibrovascular tissue, wet weight, vascularization (Hb content), macrophage recruitment (NAG activity), collagen deposition and the levels of vascular endothelial growth factor (VEGF), interleukin (IL)-1β, IL-6, IL-17, tumor necrosis factor (TNF)-α and transforming growth factor (TGF-β). These findings reflect a regulatory function of CU on multiple parameters of the main components of inflammatory angiogenesis thereby giving insight into the potential therapeutic benefit underlying the anti-cancer actions of CU (Alhaider et al., 2014). On the other hand, numerous studies have demonstrated that the anti-cancer effects of ASP could also be related to its antiangiogenic action (Huang et al., 2016; Dai et al., 2017). Other than the widely known COX-1 as a target of ASP, several other antiangiogenic targets of ASP have been studied including heparanase, angiotensin II, matrix metalloproteinase, and glucose transporter. This topic has been reviewed previously (Dai et al., 2017; Fausto de Souza et al., 2020).

In the current study, ASP was included as a well-established antiplatelet agent and used as a positive control to provide a reference point for evaluating the inhibitory effects of CU on platelet GPs expression. The comparison therefore provides a relative assessment of the inhibitory activity of CU. However, direct quantitative comparisons between CU and ASP should be interpreted with caution, as they are complex and fundamentally different substances. This observed similarity is limited to the functional outcome of inhibition of specific platelet GPs receptors particularly CD41 and CD61 and does not constitute evidence of a shared mechanism of action. Therefore, any potential relevance of the observed effects of CU to its previously reported anticancer activity remains speculative and requires further mechanistic and functional studies for confirmation.

Lastly, in all our studies we used whole raw CU, in view of the fact that it is raw urine that holds the claims of the wide range of therapeutic benefits mentioned earlier in the introduction. We are aware that CU is an excretion product and our earlier detailed proteomic analysis of CU (Alhaider et al., 2012) uncovered 1,274 peptides and 147 proteins that may have some, yet un-identified biological actions that await future characterization studies.

## 5 Study strength and limitations

The main strength of this study is that it is the first to provide molecular evidence that CU modulates platelet GP surface receptor expression, particularly CD41 and CD61 providing novel molecular insight into its antiplatelet activity. However, this study has several limitations. The flow cytometry experiments were performed using CU samples that demonstrated inhibitory effects on ADP and AA-induced platelet aggregation, which was used as an indicator of antiplatelet activity. In addition, whole fresh urine was used without identification of the active bioactive constituent(s), the platelet exposure time was relatively short, and no direct functional cancer assays were performed. Therefore, further mechanistic and *in vivo* studies are required to establish a direct relationship between these findings and anticancer activity.

## 6 Conclusion

In conclusion, the results of the current flow cytometric study have revealed that CU has significant inhibitory action on multiple platelet membrane surface GP receptors that it shares with ASP. However, ASP displayed more potent inhibitory action than CU. These findings indicate that CU possesses antiplatelet activity which may warrant further investigation as a potential contributor to its reported anticancer effects. This proposed anticancer relevance remains speculative and requires direct experimental confirmation.

## Data Availability

The raw data supporting the conclusions of this article will be made available by the authors, without undue reservation.
